# V2X Wireless Technology Identification Using Time–Frequency Analysis and Random Forest Classifier

**DOI:** 10.3390/s21134286

**Published:** 2021-06-23

**Authors:** Camelia Skiribou, Fouzia Elbahhar

**Affiliations:** COSYS-LEOST, University Gustave Eiffel, IFSTTAR, F-59650 Villeneuve d’Ascq, France; fouzia.boukour@univ-eiffel.fr

**Keywords:** Intelligent Transport Systems (ITS), Vehicle-to-Everything (V2X), signal identification, Instantaneous Frequency (IF), Singular Value Decomposition (SVD), random forest

## Abstract

Signal identification is of great interest for various applications such as spectrum sharing and interference management. A typical signal identification system can be divided into two steps. A feature vector is first extracted from the received signal, then a decision is made by a classification algorithm according to its observed values. Some existing techniques show good performance but they are either sensitive to noise level or have high computational complexity. In this paper, a machine learning algorithm is proposed for the identification of vehicular communication signals. The feature vector is made up of Instantaneous Frequency (IF) resulting from time–frequency (TF) analysis. Its dimension is then reduced using the Singular Value Decomposition (SVD) technique, before being fed into a Random Forest classifier. Simulation results show the relevance and the low complexity of IF features compared to existing cyclostationarity-based ones. Furthermore, we found that the same accuracy can be maintained regardless of the noise level. The proposed framework thus provides a more accurate, robust and less complex V2X signal identification system.

## 1. Introduction

Intelligent Transport Systems (ITS) play a significant role in improving road safety and optimizing traffic management. They rely on advanced wireless technologies to share a large amount of data collected from hundreds of embedded sensors. These information exchanges are referred to as Vehicle-to-Everything (V2X) communications, and they encompass all the communications between a vehicle and its environment [1].

Two major wireless technologies have emerged to ensure this connectivity. On one hand, the ITS-G5 has been developed by the European Telecommunications Standards Institute (ETSI), based on the IEEE 802.11p access layer. It represents an extension of the general WiFi standard optimized for vehicular environments [2]. On the other hand, the Cellular Vehicle-to-Everything (C-V2X) communications have been introduced by the Third Generation Partnership Project (3GPP) with release 14 of the Long-Term Evolution (LTE) standard [3], then expanded in release 16 with the coming of the 5G New-Radio (NR) [4].

The coexistence of ITS-G5 and C-V2X technologies will satisfy the specific requirements of transport services in terms of latency, reliability and coverage. However, several challenges will arise, since they both operate over the 5.9 GHz spectrum. One solution to facilitate this coexistence and avoid interference consists of detecting and identifying the wireless technology, then dynamically selecting the appropriate transmission channel. Therefore, the efficiency of the spectrum usage relies on the ability of the ITS station to accurately identify the received signal [5].

Signal identification has been an intensive topic of research over the last two decades. In the context of cognitive radio, the classification of digitally modulated signals has been performed in several studies [6,7,8,9,10]. The authors of [7,8] exploit the statistics derived from the instantaneous features of the incoming signals, whereas the algorithms proposed in [9,10] are based, respectively, on the statistical moments and cumulants of these signals. Another study, conducted in [11], aims to recognize single carrier modulated signals versus Orthogonal Frequency Division Multiplexing (OFDM) signals based on their cyclostationarity. This property has aroused a great deal of interest in the research community and has been employed for the identification of some standard signals as well, such as the Global System for Mobile communication (GSM) versus LTE in [12], and Worldwide Interoperability for Microwave Access (WiMAX) versus LTE in [13].

All the above-mentioned methods belong to the feature-based statistical approach. It consists of extracting explicit features from the received signal, then passing them through a classification algorithm where the decision is made based on their observed values [14]. This decision making step is mostly based on the analysis of the probability distribution function of the feature vectors or the evaluation of the Euclidean distance between their prescribed and estimated values. They have both been proven to be simple to implement, with near-optimal performance. However, they are sensitive to the noise level and/or require a priori information on the received signal [6]. Moreover, the manually set decision parameters, such as thresholds, make it challenging to systematically adapt these techniques whenever a new wireless technology emerges.

Recently, deep learning techniques have been rapidly developed and have made great strides in the signal identification field. For example, convolutional neural networks can be said to be the most popular architecture for both modulation and wireless technology recognition [15,16]. Although this approach performs well in different applications and has the advantage of simple feature pre-processing or even raw data usage, it requires large-scale training data resulting in high implementation costs [17]. Moreover, the availability of datasets for wireless communications is one of the biggest challenges for researchers. As a result, Machine Learning (ML) techniques, such as Support Vector Machine (SVM) and Random Forest, have been widely used in related studies [18,19,20]. Combined with simulation based data generation, they have shown promising results with small datasets.

The aim of this paper is thus to exploit the power of ML techniques to identify ITS-G5, LTE-V2X and NR-V2X signals in an Additive White Gaussian Noise (AWGN) channel. Wireless technology identification is a substantial field of knowledge but the vehicular context has not been considered in existing studies. The proposed approach addresses three main issues: the confusion between two close technologies such as LTE and NR; the sensitivity of accuracy to noise level; and the high computational complexity. The first step is the extraction of the feature vector by performing a time–frequency (TF) analysis on the received signal. It consists of decomposing it into Intrinsic Mode Functions (IMF) then computing their Instantaneous Frequency (IF). This combination has the power to bring out the local and unique characteristics of signals. In order to achieve the best estimation of the raw features using fewer dimensions, we also implement the Singular Value Decomposition (SVD) technique. The obtained feature vector can then be fed into any classifier for the decision making step. In this study, we used the random forest classifier because of its simplicity.

To demonstrate the superiority of its performance, we compared different classification metrics of the proposed technique with those of the SVM classifier used with Spectral Correlation Function (SCF) features [20]. The accuracy of a cyclostationarity-based technique proposed in [12] was also evaluated to show the limitations of the statistical approach.

The rest of the paper is organized as follows: Section 2 reviews some signal pre-processing techniques that are relevant to our study. An overview of the considered V2X signals is presented in Section 3 along with their physical layer parameters. Section 4 presents the proposed identification algorithm based on instantaneous frequency features and the random forest classifier. After a description of the data generation process, the obtained results are evaluated in Section 5, where the confusion matrix and other classification metrics are compared with those of the cyclostationarity-based approach. Section 6 concludes this work and proposes some future research directions.

## 2. Background

In this section, we provide a review of some common pre-processing techniques, which will be used later in the proposed algorithm and the comparative study.

### 2.1. Cyclostationarity

A signal x(t) is considered to be second order cyclostationary if its second order statistics exhibit hidden periodicities in time. Its autocorrelation function $R_{x}\left(t,\tau \right)$ can thus be expressed as [12]:

(1) $$R_{x}\left(t,\tau \right)=E\left\{x\left(t+\frac{\tau }{2}\right)x^{*}\left(t-\frac{\tau }{2}\right)\right\},$$

where τ denotes the time delay and E{.} the statistical expectation.

Applying a Fourier series expansion to Equation (1), the $T_{0}$—periodic function $R_{x}\left(t,\tau \right)$ can be represented as:

(2) $$R_{x}\left(t,\tau \right)=\sum_{\alpha }R_{x}^{\alpha }\left(\tau \right)e^{j2\pi \alpha t},$$

where $\alpha =m/T_{0},m\in Z$ are the cyclic frequencies, and the Fourier coefficients,

(3) $$R_{x}^{\alpha }\left(\tau \right)=\frac{1}{T_{0}}\int_{-T_{0}/2}^{T_{0}/2}R_{x}\left(t,\tau \right)e^{-j2\pi \alpha t}dt,$$

are referred to as the Cyclic Autocorrelation Function (CAF).

In the frequency domain, the signal x(t) is characterized by its cyclic spectrum $S_{x}^{\alpha }\left(f\right)$, also known as the Spectral Correlation Function (SCF). It is defined as the Fourier transform of the CAF and is given by [21]:

(4) $$S_{x}^{\alpha }\left(f\right)=\int_{-T_{0}/2}^{T_{0}/2}R_{x}^{\alpha }\left(\tau \right)e^{-j2\pi f\tau }d\tau .$$

The SCF cannot be directly estimated using Equation (4) because of its high computational complexity. Therefore, an efficient cyclic spectral analysis algorithm, called the Fast Fourier Transform (FFT) Accumulation Method (FAM), has been proposed to reduce this complexity. The first step consists of computing the complex demodulates $R_{T}\left(n,f\right)$ using a sliding $N^{\prime }$-point FFT as follows:

(5) $$R_{T}\left(n,f\right)=\sum_{k=-N^{\prime }/2}^{N^{\prime }/2}a\left(k\right)x\left(n-k\right)e^{-j2\pi f\left(n-k\right)T_{s}},$$

where a(n) is a Hamming window of length $T=N^{\prime }T_{s}$, and $T_{s}$ is the sampling period. In the next step, the $N^{\prime }$—point FFT is hopped over the data in blocks of size *L*. Then, the product between the complex demodulates and its conjugate is time-smoothed by a second FFT of length *P*. Hence, the SCF estimate obtained by FAM is expressed as [21]:

(6) $$S_{xT}^{\alpha_{i}+q\Delta \alpha }\left(nL,f\right)=\sum_{k}R_{T}\left(kL,f\right)R_{T}^{*}\left(kL,f\right)g_{c}\left(n-k\right)e^{-j2\pi kq/P}.$$

### 2.2. Time–Frequency Analysis

Time–frequency analysis is effective for analyzing non-stationary signals and exploring their time-varying characteristics. One common technique is the Hilbert–Huang transform, which is a two step transform proposed by Huang et al. in 1998 [22]. The first step is called Empirical Mode Decomposition (EMD), and it transfers any complex signal x(t) into the linear superposition of *K* Intrinsic Mode Function (IMF) components $c_{i}\left(t\right)\left(i=1,\ldots ,K\right)$, which contain the local characteristics of the original signal at different time scales. Therefore, the signal x(t) can be written as [23]:

(7) $$x\left(t\right)=\sum_{i=1}^{K}c_{i}\left(t\right)+r_{K}\left(t\right),$$

where $r_{K}\left(t\right)$ is the residue and represents the average trend of the signal.

The second step consists of applying the Hilbert transform to the IMF component $c_{i}\left(t\right)$ and constructing the analytic signal $z_{i}\left(t\right)$ defined as:

(8) $$z_{i}\left(t\right)=c_{i}\left(t\right)+j\tilde{c_{i}}\left(t\right)=a_{i}\left(t\right)e^{j\phi_{i}\left(t\right)},$$

where $\tilde{c_{i}}\left(t\right)$ is the Hilbert transform of $c_{i}\left(t\right)$ and is expressed as:

(9) $$\tilde{c_{i}}\left(t\right)=\frac{1}{\pi }\int_{-\infty }^{+\infty }\frac{c_{i}\left(t\right)}{t-\tau }d\tau .$$

Thus, a non-stationary complex signal x(t) can be expressed by a time-dependent function Z(ω,t) as follows [14]:

(10) Z(ω,t)=Re∑i=1Kai(t)expj∫ωi(t)dt,

where

(11) $$a_{i}\left(t\right)=\sqrt{c_{i}^{2}\left(t\right)+{\tilde{c_{i}}}^{2}\left(t\right)}$$

is the instantaneous amplitude and

(12) $$\omega_{i}\left(t\right)=\frac{d\phi_{i}\left(t\right)}{dt}\;;\;\phi_{i}\left(t\right)=\tan^{-1}\left(\frac{\tilde{c_{i}}\left(t\right)}{c_{i}\left(t\right)}\right)$$

are the instantaneous frequency and phase.

## 3. Signal Model

In this section, the OFDM signal model used for ITS-G5, LTE-V2X and NR-V2X is introduced. More specifically, we present the frame structure and the physical layer parameters of these three standards, as they have a direct impact on the time–frequency features and the periodic behavior of the signals, needed for identification purposes.

Assuming that an OFDM symbol consists of $N_{c}$ subcarriers at frequencies $f_{0}$, $f_{1}$, …, $f_{N_{c}-1}$ separated by Δf, the baseband equivalent transmitted signal x(t) is given by:

(13) $$x\left(t\right)=a\sum_{k}\sum_{n=0}^{N_{c}-1}s_{n,k}e^{j2\pi f_{n}\left(t-T_{cp}-kT_{s}\right)}g\left(t-kT_{s}\right),$$

where $a=\sqrt{E_{s}/N_{c}}$ is the amplitude factor with $E_{s}$ representing the signal power. $s_{n,k}$ denotes the transmitted symbol within the *n*-th subcarrier and the *k*-th symbol period. These symbols are assumed to be independent and identically distributed (i.i.d) random variables with values drawn from an *M*-ary Quadrature Amplitude Modulation (QAM) constellation. $T_{s}$ is the symbol period given by $T_{s}=T_{u}+T_{cp}$, with $T_{u}=1/\Delta f$ denoting the useful symbol duration and $T_{cp}$ the length of the Cyclic Prefix (CP). The function t↦g(t) is the pulse shaping filter.

Therefore, the baseband-equivalent received signal affected by the AWGN channel is expressed as:

(14) $$y\left(t\right)=a\sum_{k}\sum_{n=0}^{N_{c}-1}s_{n,k}e^{j2\pi f_{n}\left(t-T_{cp}-kT_{s}\right)}g\left(t-kT_{s}\right)+n\left(t\right),$$

where n(t) denotes the zero mean white Gaussian noise of variance $\sigma_{n}^{2}$.

### 3.1. ITS-G5

The physical layer of ITS-G5 is based on IEEE 802.11p, a modified version of the IEEE 802.11a standard. The main difference is that the subcarrier spacing and bandwidth are halved, which results in a symbol duration twice as long. The cyclic prefix duration is also doubled, which allows us to compensate for larger delay spreads and makes it more suitable for vehicular environments [24].

The IEEE 802.11p frame consists of three main fields. The first field lasts 32 µs and is called the preamble. It is used for channel assessment before transmission and for signal detection at the receiver side. The second element of the frame is the signal field and consists of one OFDM symbol. It is intended to indicate the data rate, packet length and modulation scheme of the transmitted signal. The last element is the data field, which has a variable number of OFDM symbols. It contains data, tail and padding bits [2].

For OFDM transmission, a total of 64 subcarriers is used. The 0th and the central 11 subcarriers are null. Those with indices 7, 10, 44 and 58 are occupied by pilot symbols, and the remaining 48 are used for carrying data [25]. The OFDM symbol lasts 8 µs and the subcarrier spacing is 156.25 kHz, leading to a raw bandwidth of 10 MHz. ITS-G5 supports a wide range of modulation schemes, from Binary Phase Shift Keying (BPSK) to 64-QAM [26].

### 3.2. LTE-V2X

LTE-V2X supports 10 MHz and 20 MHz channels. Each channel is divided into subframes, Resource Blocks (RBs) and subchannels. A subframe is 1ms long, as is the transmission time interval. It consists of 14 OFDM symbols with a normal cyclic prefix. Those with indices 3, 6, 9 and 12 are used for channel estimation and carry Demodulation Reference Signals (DMRS); the last symbol is used as a guard period for Tx-Rx timing adjustment, and the remaining are the actual data symbols [4].

A resource block represents the smallest unit of frequency resources and is made up of 12 subcarriers of 15 kHz spacing (total of 180 kHz). A combination of RBs in the same subframe is referred to as a subchannel in LTE-V2X, and each subchannel may have a different number of RBs [27].

Within the same subframe, a subchannel is used to transmit Transport Blocks (TB) over the physical sidelink shared channel, and Sidelink Control Informations (SCI) over the physical sidelink control channel. A TB contains user data information and must be transmitted with its associated SCI. An SCI carries information, including the modulation and coding scheme, which is crucial to decode user data. It is always sent using the Quadrature Phase Shift Keying (QPSK) modulation scheme, whereas TB can also support the 16-QAM modulation scheme [28].

### 3.3. NR-V2X

The 3GPP Release 16 defines the first specifications for the NR-V2X sidelink. It supports the same numerology and frequency bands as the NR Uplink/Downlink, but only the CP-OFDM waveform is used. A channel bandwidth up to 100 MHz is allowed in the first Frequency Range (FR1) with a subcarrier spacing ranging from 15 kHz to 60 kHz, against 400 MHz in the second one (FR2), where the subcarrier spacing parameter takes the maximum value of 120 kHz. Four modulation schemes are available, namely QPSK, 16-QAM, 64-QAM and 256-QAM [4].

The frame structure of 5G-NR allows flexible configurations to enable novel V2X use cases. Similar to LTE, the frame length is fixed to 10 ms and is divided into ten equally sized subframes. The subframe is further subdivided into slots, depending on the used numerology. Each slot has 14 OFDM symbols, forming a typical transmission unit [29].

Unlike LTE, the reference signals of 5G-NR are time and frequency configurable. Indeed, the DMRS, used by the receiver to produce channel estimates for data demodulation on the physical channels, is specified with a structure that has a front-load DMRS mapped in the front part of the data channel, as well as the additional mapping of 0–3 symbols of additional DMRS. Each design aims to find the best tradeoff between channel estimation accuracy improvement and DMRS overhead reduction [30].

Very low latency and minimum interference with other signals is achieved with mini slot transmission. It consists of transmitting the physical channel and its DMRS over a fraction (2, 4, or 7 symbols) of the slot.

## 4. Signal Identification

**Figure 1:**
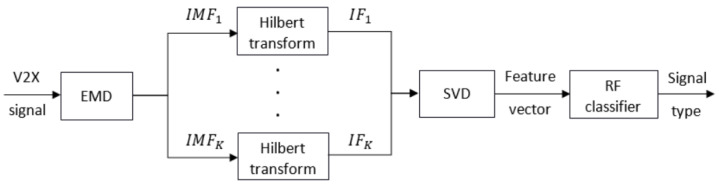
Pipeline of the proposed signal identification system.

In this section, we detail the proposed algorithm steps for identifying the communication signal received by an ITS station. The pipeline is depicted in Figure 1. First, we describe the feature extraction process and the SVD technique, then we present the random forest classifier used for the decision making, and finally we define some classification metrics for the performance evaluation.

### 4.1. Feature Vector Extraction

The feature extraction procedure starts by applying empirical mode decomposition to the received signal y(t) using Equation (7). The obtained IMF components represent the original signal from high frequency to low frequency in different frequency bands. In addition, the first few IMFs are significant as they have the largest energy and contain the most important information from the I/Q signal. Therefore, the instantaneous frequencies of the prior *K* IMFs are then extracted using Equation (12). The value of *K* depends on the signal length and complexity. In practice, it is usually set between three and five [31,32].

Given *N* is the length of the signal of interest y(t), the number of elements of the feature vector made up of *K* IFs is equal to KN, leading to a high-dimensional dataset. Consequently, dimensionality reduction is required in order to reduce the overall execution time and thus improve the classification model performance. In this context, singular value decomposition might be the most popular and efficient dimensionality reduction technique in machine learning. It comes from the field of linear algebra and consists of decomposing an *m* × *n* matrix M into three matrices U, Σ and V as follows:

(15) $$M=U\Sigma V^{T},$$

where U and V are two orthogonal matrices of dimensions *m* × *m* and *n* × *n*, respectively, and Σ is an *m* × *n* diagonal matrix. The diagonal entries $\sigma_{i}\;\left(i=1,\ldots ,r\right)$ of Σ are positive real values listed in descending order. They represent the singular values of M, while *r* is equal to its rank [33].

By applying the SVD algorithm to the previously constructed feature vector, the most important structure in the raw data is preserved whilst reducing its dimension to 1 × *K*. Therefore, the obtained time–frequency feature vector that will be used for signal identification is given by:

(16) $$S={\left[\sigma_{1}\left(\omega \right),\ldots ,\sigma_{K}\left(\omega \right)\right]}^{T},$$

where $\sigma_{i}\left(\omega \right)$ is the singular value related to the instantaneous frequency of the *i*-th IMF.

### 4.2. Random Forest Classifier

After the feature vector is generated, it is fed into the classification model to determine the class to which the signal belongs. The model selection should take into consideration both accuracy and complexity. The random forest classifier has been reported to be one of the most effective off-the-shelf methods in machine learning, working well for a wide range of problems [34].

This method consists of building an ensemble (forest) of decision trees. Each tree provides a classification result and the forest chooses the class that has the highest votes as the overall output [35]. Random Forest increases the diversity of the trees by making them grow from different training data subsets created through bootstrap aggregating (bagging) [36]. The implementation steps of a random forest classifier can thus be summarized as follows [23]:

- Building the individual trees of the forest using algorithms such as C4.5 or CART.
- Sampling randomly the original training dataset without deletion of the selected data in order to create an in-bag subset for each tree.
- Selecting randomly a set of features to construct the nodes and leaves of each tree.
- Selecting the root node of the tree, which represents the attribute (feature) with the highest Information Gain (IG).
- Splitting the training data at the root node into subsets for every possible value of the attribute. Then, at each node, the splitting is conducted if the IG is positive; otherwise the node becomes a leaf node. The information gain of splitting the training dataset (*Y*) into subsets ($Y_{i}$) is given by:
  (17) $$IG=-\sum_{i}\frac{\mathrm{size}(Y_{i})}{\mathrm{size}(Y)}E\left(Y_{i}\right)\;;\;E\left(Y_{i}\right)=-\sum_{j=1}^{J}p_{j}\log_{2}\left(p_{j}\right),$$
  where *J* is the number of signal classes and $p_{j}$ the proportion of the class *j* in the subset $Y_{i}$.
- Repeating this process of tree growing at each node using the subset that reaches the branch and the remaining attributes until all attributes are selected. The most occurring signal class that reached that node is the classification output of the tree.

It is worth mentioning here that injecting randomness in both bagging and feature selection strategies increases the stability and the accuracy of classification, decreases the sensitivity to noise in the data, and minimizes the correlation among features [35].

### 4.3. Classification Metrics

To assess the performance of the proposed technique, we need to define the three metrics mainly used for classification problems, which are precision (Π), recall (Ψ) and $F_{1}$-score. The precision gives an idea of how many of the results determined as positive are actually positive. The recall is a measure denoting how many true positives are correctly identified. The $F_{1}$–score is an overall measure of the accuracy of the classifier and represents the harmonic average of precision and recall. These metrics are given by [21]:

(18) $$\Pi =\frac{\xi }{\xi +\upsilon },\;\Psi =\frac{\xi }{\xi +\mu },\;F_{1}-\mathrm{score}=2\times \frac{\Pi \times \Psi }{\Pi +\Psi },$$

where ξ, υ and μ denote the numbers of true positives, false positives and false negatives, respectively.

In addition, we define the accuracy *P* as the measure of how well accurate recognition can be performed by the classifier. It is given by:

(19) $$P=P({\hat{\chi }}_{l}=\chi_{l}),\;l=0,1,2,$$

where $\chi_{l}$ and ${\hat{\chi }}_{l}$ denote the label arrays of the received and the predicted signals, respectively. While the index l=0,1,2 represents the label of the classes ITS-G5, LTE-V2X and NR-V2X, respectively.

## 5. Performance Evaluation

The aim of this section is to evaluate the performance of the proposed identification technique and to compare it with that of the existing cyclostationarity-based ones. So, we first describe the process to generate the vehicular signals as well as the resulting feature vectors that are used to train and test the two classifiers, then we present the simulation results comparing the performance metrics of both approaches.

### 5.1. Dataset Generation

**Figure 2:**
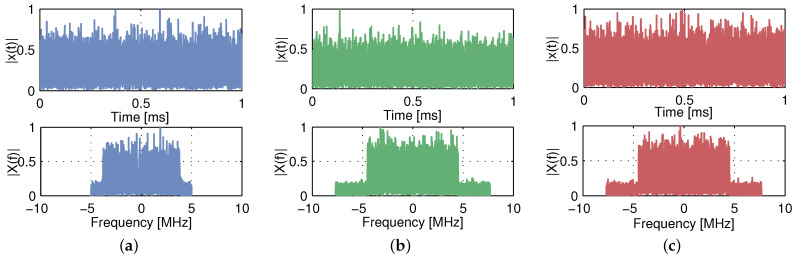
Time and frequency domain representation of received (**a**) ITS-G5 (**b**) LTE-V2X and (**c**) NR-V2X signals at SNR = 10 dB.

The vehicular communication signals dataset used in this study is a synthetically generated dataset obtained using MATLAB [37]. It contains feature vectors extracted from ITS-G5, LTE-V2X and NR-V2X signals along with their respective labels. For each label (signal type), the simulations are performed at 15 different Signal-to-Noise Ratio (SNR) levels ranging from −10 dB to 18 dB, and each level consists of the same number of signals. As a result, the dataset covers, for the three wireless technologies, a total of 4500 signals, whose parameters are summarized in Table 1 and which stem from the possible configurations previously described in Section 3. An example of each signal type received at SNR = 10 dB is depicted in Figure 2 in both time and frequency domains.

For the feature extraction step, we consider two feature types. The first one is the SCF of the generated signals, estimated by Equation (6) and used as an input to the SVM classifier [20]. The length of the feature vector is set here, as in the original study, to 1 × 16,385, leading to a dataset dimension of 4500 × 16,386. The second feature is the singular values of IFs, given by Equation (16) and fed into the random forest classifier, as seen in Figure 1. We set *K*, the number of IMFs, to the lowest value that can be considered leading to a feature vector of three elements and a dataset dimension of 4500 × 4.

### 5.2. Simulation Results

#### 5.2.1. Data Analysis

To better understand the resulting datasets, we need to visualize the feature vectors of the three considered signal types in two-dimensional space. For ease of plotting, the t-Distributed Stochastic Neighbour Embedding (t-SNE) technique is used. It is a method for visualizing high-dimensional data by giving each sample a location in a two or three-dimensional space, whilst preserving distances between samples [38].

**Figure 3:**
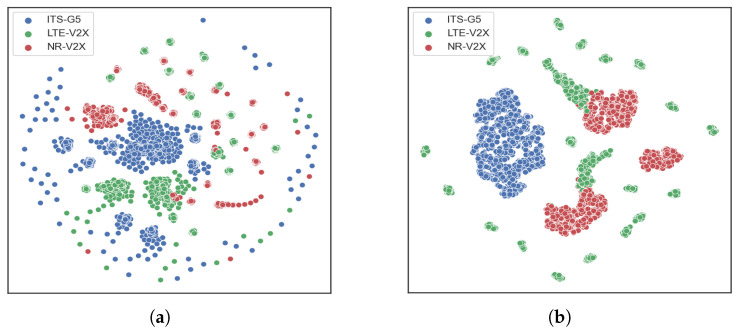
t-sne representation of (**a**) SCF and (**b**) IF features.

As can be seen in Figure 3, the t-sne representation of both datasets clusters the three signal types into distinct groups in space. However, by comparing the two graphs, we can first observe that the SCF feature makes the separation harder and will consequently require a more complex classifier such as SVM. Moreover, the samples not belonging to any of the formed clusters or those superimposed on each other may increase the confusion among signals, unlike the IF feature samples in which almost no confusion can be seen. Therefore, the proposed feature vector allows us to address the first issue of signal identification, which consists of reducing the confusion between signals sharing the same PHY layer parameters. More in-depth analysis is required to explore these preliminary results in greater detail, which will be performed in the next subsection through confusion matrices and the previously defined classification metrics.

#### 5.2.2. Performance Analysis

The proposed approach, consisting of IF features combined with the random forest classifier, is evaluated in tandem with that based on SCF features and the SVM classifier. Each of the datasets is shuffled and split into training and test sets containing 3000 and 1500 samples, respectively. Then, the input data is normalized and scaled, which is a crucial step to alleviate the effect of SNR variations, especially for distance-based classifiers like SVM. Implementation and evaluation are conducted in the open source Scikit-learn software library [39].

**Figure 4:**
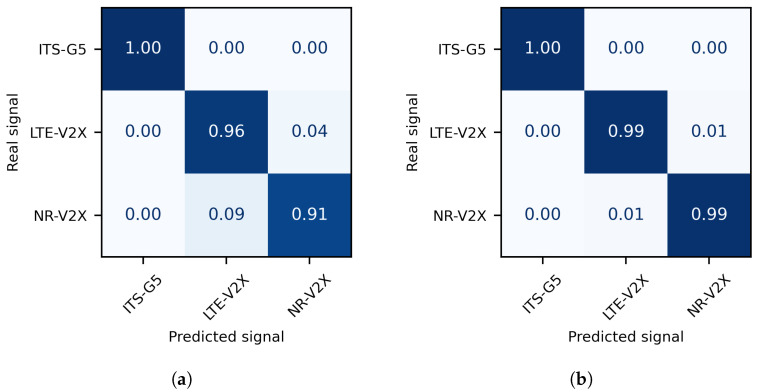
Confusion matrix of (**a**) SCF with SVM and (**b**) IF with random forest techniques.

The confusion matrices of the two considered techniques are depicted in Figure 4. They show that the SCF features provide a slightly worse performance compared to the IF features when dealing with LTE-V2X and NR-V2X signals. Indeed, the two technologies share many PHY layer parameters, as previously seen in Section 3. Since SCF reveals the hidden periodicities within the signals, which are caused by the symbol period and cyclic prefix duration among others, their similarity decreases the distance between samples and prevents the SVM algorithm from correctly identifying those signals.

Their identification rates are 96% and 91%, respectively. The difference between these two rates flows from the fact that the NR-V2X standard has more configurations, and thus more dispersed SCF values, than the LTE-V2X standard. Therefore, the boundary placed by the SVM classifier to identify the NR-V2X signals is less accurate than that of the LTE-V2X signals. On the other hand, the random forest classifier increases the identification rate to 99% for both signals because the confusion between them has been decreased by using IF features. Their relevance comes from the power of intrinsic mode functions and instantaneous frequency to bring out the local time–frequency characteristics of the signals.

When it comes to the 802.11p technology, its unique characteristics make it more distinguishable, and both cyclostationarity and time–frequency based features can be used to identify ITS-G5 signals with an accuracy of 100%.

The precision, recall and F1–score of the three signal types and both techniques are summarized in Table 2. A simple comparison shows that the classification results are in line with the previous t-sne analysis. However, they only represent the global performance of the algorithms within a wide range of SNRs and do not really reflect the impact of this parameter on the identification accuracy.

**Figure 5:**
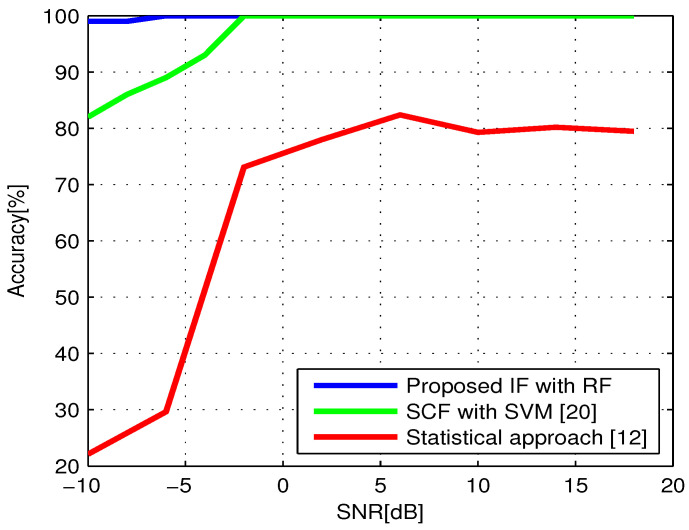
Classification accuracy with respect to SNR.

In order to investigate this relationship, the two classifiers are trained and tested on signals of each SNR level separately. Figure 5 depicts the accuracy variation of both techniques, along with that obtained by implementing the classification algorithm in [12], taken as an example of a comparison with the statistical approach.

The results show that the cyclostationarity-based features are more sensitive to the noise level than the proposed IF features. For instance, the SVM classifier exceeds 90% accuracy at −4 dB, then gives the best performance, 100%, at −2 dB and remains constant until 18 dB. This behavior can be explained by the decrease of SCF amplitudes at low SNRs. Therefore, the difference between the cyclostationarity properties of C-V2X signals that have similar configurations becomes more difficult to discern, leading to a higher number of classification errors. Similarly, the statistical approach, based on a comparison between the CAF estimates of the considered signals at their cyclic frequencies and a threshold value determined by setting the probability of false alarm to 0.1, shows the poorest performances and its accuracy strongly depends on the SNR. It achieves a maximum value of 82% at 6 dB then fluctuates around 80% for the higher SNR regimes. On the other hand, the accuracy of the proposed model is almost stable at 100% for all SNR values due to the insensitivity of the time–frequency features to noise level.

This discussion demonstrates why the proposed ML approach represents a better choice for fulfilling the high requirements of vehicular applications in terms of accuracy, and why cyclostationarity-based features cannot maintain the same level of performance regardless of SNR value.

#### 5.2.3. Complexity Analysis

So far, the proposed technique outperforms the SCF with SVM technique in terms of classification accuracy. However, the computational complexity is another important parameter that needs to be explored in order to make the optimal choice of features.

By applying the FFT accumulation method described in Section 2, the computational complexity of SCF estimation is given by $O(2N\left[4+2\log_{2}\left(N^{\prime }\right)+4N+2N^{\prime }+N^{\prime }\log_{2}\left(\frac{4N}{N^{\prime }}\right)\right])$, where *N* is the signal length [20]. By keeping the highest order terms of the Big-O notation, the overall time complexity is $O(N^{2})$. Therefore, the SCF combined with SVM technique is computationally expensive although it has a relatively good classification performance.

On the other hand, the extraction of IF features involves empirical mode decomposition, Hilbert transform and singular value decomposition. The computational complexity of the three algorithms is O(KN) [40], $O(N\log_{2}\left(N\right))$ [41] and $O(K^{2}N)$ [42], respectively. Therefore, the overall time complexity of the proposed technique is as low as $O(N\log_{2}\left(N\right))$. Moreover, the dataset size of 4500 × 4 significantly decreases the training processing time of the classifier.

## 6. Conclusions

In this study, an ML-based technique for the identification of V2X communication signals without any prior information is proposed. It combines the use of robust features based on time–frequency analysis along with the random forest classifier.

First, we present the model of the three considered signals as well as their physical layer parameters. A comparison of these parameters shows that LTE-V2X and NR-V2X have similar properties, in particular those related to the periodicity of signals. Their instantaneous frequency is thus extracted to distinguish between them, then passed through the SVD algorithm to reduce their dimensionality.

By implementing the random forest classifier, the results show the effectiveness of our approach and the superiority of IF as a distinctive feature when compared to the cyclostationarity-based feature utilized in many existing studies. Moreover, comparative analysis with the statistical approach indicates that the latter is not suitable for identifying signals that have similar CFs, and that it is highly dependent on the SNR level.

In subsequent studies, the performance of the proposed identification technique can be explored on vehicular signals affected by multi-path fading channels. Furthermore, the proposed technique can also be used for real-world applications such as dynamic spectrum access or jamming signals detection.

## Figures and Tables

**Table 1:** PHY layer parameters of generated vehicular communication signals.

	ITS-G5	LTE-V2X	NR-V2X
Bandwidth	10 MHz	{10, 20} MHz	{10, 20, 50} MHz
Subcarrier spacing	156.25 kHz	15 kHz	{15, 30} kHz
FFT size	64	{1024, 2048}	{512, 1024, 2048}
CP size	16	{72, 144}	{36, 72, 144}
QAM order	{4, 16, 64}	{4, 16}	{4, 16}

**Table 2:** Performance metrics of random forest and support vector machine classifiers.

Model	Signal	Precision	Recall	F1–Score
Proposed IF with RF	ITS-G5	1	1	1
	LTE-V2X	0.99	0.99	0.99
	NR-V2X	0.99	0.99	0.99
	Average	0.99	0.99	0.99
SCF with SVM [20]	ITS-G5	1	1	1
	LTE-V2X	0.91	0.96	0.94
	NR-V2X	0.96	0.91	0.93
	Average	0.96	0.96	0.96

## Data Availability

The data presented in this study are available on request from the corresponding author.

## Abbreviations

The following abbreviations are used in this manuscript:

AWGN	Additive White Gaussian Noise
BPSK	Binary Phase Shift Keying
CAF	Cyclic Autocorrelation Function
CP	Cyclic Prefix
DMRS	Demodulation Reference Signal
EMD	Empirical Mode Decomposition
FAM	FFT Accumulation Method
FFT	Fast Fourier Transform
GSM	Global System for Mobile communication
IF	Instantaneous Frequency
IMF	Intrinsic Mode Function
ITS	Intelligent Transport Systems
LTE	Long-Term Evolution
ML	Machine Learning
NR	New Radio
OFDM	Orthogonal Frequency Division Multiplexing
QAM	Quadrature Amplitude Modulation
QPSK	Quadrature Phase Shift Keying
RB	Ressource Block
SCF	Spectral Correlation Function
SCI	Sidelink Control Information
SNR	Signal to Noise Ratio
SVD	Singular Value Decomposition
SVM	Support Vector Machine
TB	Transport Block
TF	time–frequency
t-SNE	t-Distributed Stochastic Neighbour Embedding
V2X	Vehicle-to-Everything
WiMAX	Worldwide Interoperability for Microwave Access
